# Prognostic Factors and Metastatic Patterns in Primary Myxoid/Round-Cell Liposarcoma

**DOI:** 10.1155/2011/538085

**Published:** 2011-11-28

**Authors:** J. Haniball, V. P. Sumathi, L.-G. Kindblom, A. Abudu, S. R. Carter, R. M. Tillman, L. Jeys, D. Spooner, D. Peake, R. J. Grimer

**Affiliations:** The Royal Orthopaedic Hospital Oncology Service, Royal Orthopaedic Hospital NHS Foundation Trust, Bristol Road South, Birmingham B31 2AP, UK

## Abstract

*Background*. This study aimed to investigate prognostic factors for patients with myxoid/round-cell liposarcoma (MRCLS), in particular the significance of the round cell component, and to identify metastatic patterns as well as possibly suggest a suitable strategy for followup. *Methods*. Clinical, morphologic, and follow-up data from 160 patients with MRCLS was reviewed and statistically analysed. *Results*. Of 130 tumours with the round cell component evaluated, 61 had no round cell component, 27 had <5% round cell component, and 42 had >5%. All patients underwent surgical excision, 15 requiring amputation. 107 patients received adjuvant radiotherapy. Local recurrence occurred in 19 patients (12%), predominantly in patients with marginal or intralesional margins and a round cell component. Overall disease specific survival was 75% at 5 years and 56% at 10 years and was related to the proportion of round cell component. Of 52 patients who developed metastases, 38 (73%) had purely extrapulmonary metastases. We could not identify any factors predicting the site of metastases developing. *Conclusions*. The occurrence of any round cell component is the most important adverse prognostic factor for patients with MRCLS; patients with >5% round cell component are at higher risk of local recurrence, metastasis and tumour-related death and should be considered for adjuvant radiotherapy and possibly chemotherapy. The best method of monitoring extrapulmonary metastases remains to be established.

## 1. Introduction

Liposarcomas, which constitute a family of malignant mesenchymal neoplasms showing adipocytic differentiation, are the most common soft tissue sarcoma in adults [1]. Four main forms of liposarcoma are recognized: atypical lipomatous tumour/well-differentiated liposarcoma, dedifferentiated liposarcoma, myxoid/round-cell liposarcoma, and pleomorphic liposarcoma [2]. Myxoid/round-cell liposarcoma (MRCLS) is the second most common subtype of liposarcoma. Histologically MRCLS shows a continuous spectrum of lesions which at one end of the spectrum exhibit a striking resemblance to developing foetal fat with ample lipoblast differentiation and at the other end of the spectrum show poorly differentiated round cell features with at best inconspicuous lipoblast differentiation. The continuous morphologic spectrum, the shared clinical characteristics, and the common chromosome translocation t(12; 16), resulting in the fusion of the TLS (FUS) and CHOP (DDT3) genes seen in most of these tumours, clearly indicate that MRCLS represents a distinct family among liposarcomas [3–6]. 

The subclassification of liposarcomas has a dramatic prognostic significance; while tumours in the spectrum of atypical lipomatous tumours/well-differentiated liposarcoma only carry a risk of local recurrence and possible dedifferentiation, the pleomorphic liposarcomas are high-grade malignancies with a very high risk of metastatic disease [2]. MRCLS carries an intermediate risk with approximately one-third of patients developing metastases and eventually dying of their tumours [7–10]. Another feature distinguishing MRCLS from the other types of liposarcoma and most other soft tissue sarcomas is its tendency to metastasize to other soft tissue sites, including the trunk and extremities, the retroperitoneum, the chest wall, the pleura, and the pericardium [11–13]. Factors found to influence the prognosis of MRCLS include the patient's age, tumour size, tumour grade, tumour depth, and the surgical margins achieved [14–16]. When compared to other types of liposarcoma, MRCLS shows an unusually high response rate to both adjuvant radiotherapy and chemotherapy [17–19]. Morphologic factors reported to be of prognostic significance in MRCLS include tumour necrosis, grading based on differentiation, necrosis and mitotic rate, proliferation index (MiB-1, Ki-67 immunostain), and P53 overexpression [8–10, 14]. Of particular interest is the fact that the rare, purely round cell liposarcoma has a clearly worse prognosis than the purely myxoid liposarcoma [15]. Also, smaller components of round cells have been shown to influence the prognosis. Kilpatrick et al. have suggested a three-tiered system (0–5%, 5–25%, >25%) while Antonescu et al. have proposed a two-tiered system (< or >5% round cell component) [8, 16]. Others have found that any round cell component is associated with a worse outcome [14]. 

The aim of this retrospective analysis was to attempt to confirm or refute the previously identified clinicopathologic prognostic factors for patients with MRCLS and in particular to analyse the significance of morphologic features based on a large single centre series with long-term followup. We also wished to investigate further the propensity for MRCLS to metastasize outside the lungs in order to establish, if possible, a reasonable followup policy for patients with this tumour.

## 2. Materials and Methods

We retrospectively reviewed the records and histopathology of all newly diagnosed patients with morphologically proven MRCLS treated at our unit from January 1987 to December 2005 (thus, allowing a minimum two-year followup). Data was collected from a prospective database which has collected information on patients, tumours, treatment, and outcome since 1986. Patients were excluded from the study if they were referred for a second opinion and were not treated at our centre or if they had metastases or local recurrence on presentation. We included patients who had undergone a previous biopsy or inadvertent excision. Out of a total of 190 patients with a diagnosis of MRCLS in the 18-year period under investigation, twelve patients with metastases at the time of diagnosis and eighteen patients referred with local recurrence after previous treatment were excluded. All the 12 patients with metastases at presentation (who were excluded from the study) died within three years. The remaining 160 patients were followed up for a minimum of two years (mean 7.2 years, median 4.6 years) as of December 2007. 

The data collected from the database included a wide range of variables including patient characteristics (age, sex), tumour characteristics (site, size, duration of symptoms, depth, stage, histopathological variables), management (biopsy type, operation type and margins achieved, use of adjuvant therapy), and followup (development of local recurrence, distant metastasis, and survival). For patients who had not been seen in the past year or who had been discharged, followup was carried out using the NHS tracking system linked to the cancer registry to identify if the patients had died and their cause of death. The tumour locations were classified as central if the tumour was located in the trunk (proximal to hip and shoulder joint), upper extremity if it was beyond the shoulder joint, and lower extremity if it was beyond the hip joint. Tumour size was defined as the diameter of the primary tumour in maximum dimension as reported at the time of initial surgical resection. Tumour depth was defined in relation to the superficial investing fascia. All retroperitoneal, abdominal, or pelvic locations were considered deep. 

The diagnosis of MRCLS was based on histopathologic examination of needle trucut biopsy, open incisional/excisional biopsy, or a previous inadequate excision of the tumour in the mistaken belief of a benign lesion at an outside centre. In the event of inadvertent excision carried out in an from an outside centre, the patient was referred to our unit for further management. Our policy in these cases was to restage the patient and usually to carry out a wide re-excision [20].

**Figure 1:**
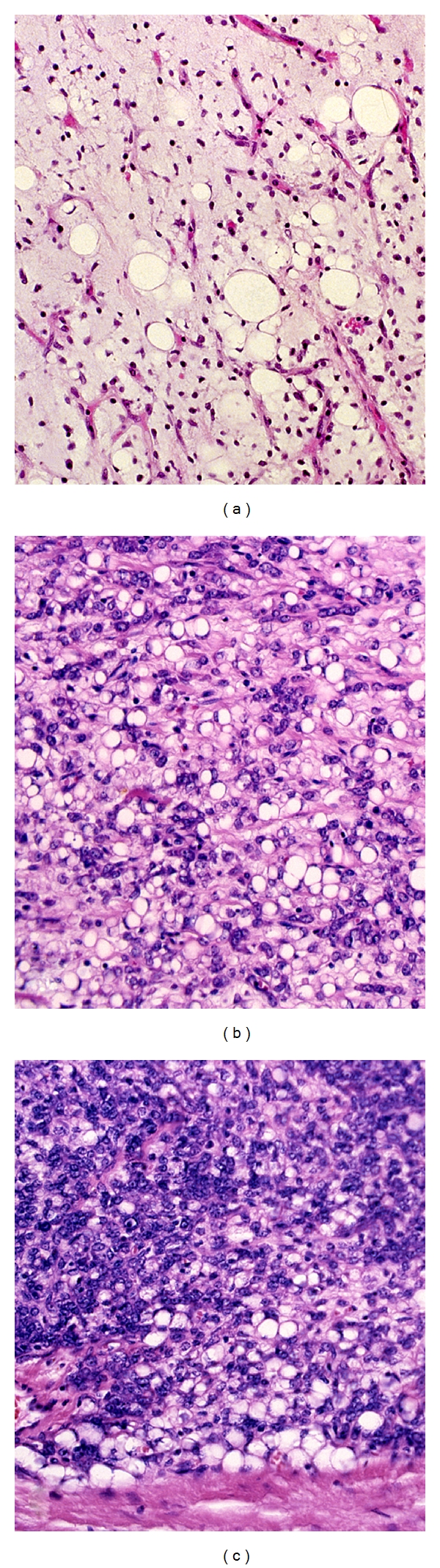
Paucicellular myxoid liposarcoma with obvious lipoblastic differentiation (a), cellular variant of myxoid liposarcoma (b), and typical round cell component (c). (Hematoxylin-eosin).

 Histologically the MRCLS resection specimens were classified as of purely myxoid type or of myxoid type with a round cell component. Tumours within the purely myxoid subgroup showed a wide morphologic spectrum in terms of cellularity and lipogenic differentiation but were all characterized by bland, uniform, fusiform, or round cells in a myxoid background and a prominent delicate, plexiform capillary network. Round cell components were either seen as sharply demarcated nodules or as a gradual transition from cellular areas of myxoid liposarcomas. Round cell components were defined as highly cellular areas with back to back primitive round cells with increased nucleocytoplasmic ratio and usually prominent nucleoli. Such areas lacked the myxoid background, and the capillary network could usually not be discerned (Figure 1). In purely myxoid liposarcomas, the most cellular areas were selected, and in tumours with a round cell component the areas were recorded. 

According to the Trojani grading system, purely myxoid liposarcomas are grade 2, while tumours with a prominent round cell component are grade 3 [3, 21]. The round cell component in myxoid liposarcoma was initially evaluated as a continuous variable but was later divided into three categories on the basis of percentage of round cell component: (1) 0% round cell, (2) <5% round cell, (3) >5% round cell. Histologic material was only available for review in 130 of the 160 patients (81%). Since all of these cases had been externally reviewed in 1993, we included these cases in the statistical analysis of clinical factors [22].

Following diagnosis and staging, the primary management aimed to excise the tumour with wide margins wherever possible [23]. When the tumour was adjacent to a critical structure (e.g., nerve, bone, blood vessel), a planned marginal margin was usually accepted [24]. Amputation was reserved for large tumours which could only be surgically removed safely by such a procedure. Postoperative radiotherapy (usually 60 Gy) was given to most patients with large (>5 cm), deep tumours with marginal margins of excision. Chemotherapy was only given to patients with metastatic disease. Patients were followed up 3 times monthly for two years, 6 times monthly until 5 years, and then annually for 8 years. They had a routine chest radiograph at each clinic visit but had no other routine imaging unless the patient or reviewing doctor was concerned.

### 2.1. Statistical Methods

Survival time was taken from the date of diagnosis to the last date when the patient was documented to be alive or the date of death. Differences between groups were analysed using the chi-squared test for discrete variables or use of the two-sample *t*-test or Mann-Whitney test for continuous variables. Survival analysis was done using the Kaplan-Meier survivorship. The Cox proportional hazards regression model was used to assess the effect (hazard ratio (HR)) on the primary outcome of age at diagnosis, location of tumour (central versus peripheral), size, depth, round cell percentage, type of surgery (limb salvage versus amputation), and margins achieved (intralesional, marginal, or wide). 

For quantitative variables (continuous variables), we report the median, first, and third quartile values. Categorical variables are reported as counts. All analyses were performed with Statview [25]. All tests were two sided, with a significance level of 0.05. When patients were grouped for more detailed analysis, this has been clearly specified in the text and the results.

## 3. Results

### 3.1. Clinicopathologic Factors

160 patients were included in the study; 99 were males (62%) and 61 females (38%). The age range was 12 to 92 years (mean 48.6 years; median 47 years).The most common presenting complaint was development of a painless soft tissue mass with duration of symptoms ranging from 1 week to 15 years (median 6 weeks, interquartile range 50 weeks) prior to diagnosis. The anatomic distribution of the primary tumours was as follows: 148 tumours occurred in the lower extremities (95 in the thigh, 23 in the lower leg, 14 in the buttock or hip, 8 in the foot or ankle, and 8 in the knee or popliteal fossa). 7 tumours occurred in the upper extremities (5 in the upper arm or shoulder; 1 in the forearm, and 1 in the hand). 5 tumours were central (3 in the lower back and 2 in the pelvis). The tumour size ranged from 1 to 36 cm in the greatest dimension (mean: 11 cm). Out of the 155 extremity tumours, only 31 were superficial to the fascia. The superficial tumours were significantly smaller than the deep tumours (*P* < 0.0001) with deep tumours averaging 13.3 cm and superficial 8.2 cm. There was no significant difference in size between upper- and lower-limb tumours.

All of the 130 histologically reviewed primary tumours had predominantly or partly classical features of myxoid liposarcoma. 61 (47%) were entirely of myxoid type while 69 (53%) had a round cell component present. 27 tumours (21%) had a round cell component of 1–5% while 22 tumours (17%) contained a round cell component of 6–25% and 20 tumours (15%) had a round cell component of >25%. Of the latter group, 8 were almost entirely of round cell type (>80% round cell component). There was no difference in tumour size, depth, or duration of symptoms between tumours with and without a round cell component. However, all of the tumours with a round cell component occurred in the lower limb or trunk. 

All patients underwent surgical excision of the primary tumour with 141 undergoing excision alone, 3 having excision and split skin grafting, 15 undergoing amputation, and 1 patient undergoing excision and endoprosthetic replacement for a tumour that encircled and invaded the proximal femur. The margins were evaluated both grossly and microscopically in all dimensions. 55 patients had a wide excision, 86 a marginal excision, and 19 an intralesional or contaminated excision. 

Postoperative radiotherapy was given to 107 patients (67%). Radiotherapy was used increasingly over the period of this study but was generally not used in patients who had undergone an amputation or in those with small tumours having undergone a wide excision. No patient received adjuvant chemotherapy at the time of initial treatment. The median followup for the 100 survivors was 6.7 years (range 7 months to 20 years) and was 3 years (range 1 month to 14.5 years) for the 60 who died. Only 46 of the deaths were tumour related (mean 3.4 years following diagnosis) with the other 14 dying of a multitude of other causes (5 of other cancers, 5 of cardiovascular disease, and 4 of other causes) at a mean of 5.6 years following diagnosis (at an age ranging between 60 and 98, mean 76 years).

### 3.2. Local Recurrence

19 patients (12%) developed a local recurrence at the primary site at a mean of 20 months. The overall risk was 12.9% at 5 years and 14.3% at ten years. The risk was directly related to the margins achieved with the risk being 32% for patients with intralesional excisions, 6% for marginal excisions, and 14% for those with wide excisions. The risk of local recurrence was also related to the round cell component, being 26% in those with >5% round cells. Because radiotherapy was used on an individual case selection basis, it was difficult to assess its benefit. Local recurrence occurred in 9 of the 87 patients with marginal or intralesional excisions who received postoperative radiotherapy (10%) compared with 2 of the 18 who did not have radiotherapy (11%). The risk of local recurrence rose by a factor of 3.3 for tumours with a round cell component >5% whether they had radiotherapy or not (Table 1). 

Of the 19 patients who developed local recurrence, 8 (42%) were found to have metastases synchronously and 5 (26%) developed metastases later (at a mean of 17 months), whilst 6 patients (32%) have not developed metastases (at a mean of 42 months since the development of local recurrence). 12 of the 19 with local recurrence subsequently died of metastases. 11 of the patients developed local recurrence within 18 months, and all but one of these patients subsequently died of metastases (91%). Of the 8 patients who developed local recurrence after 18 months, only 2 have since died of disease (25%) (*P* = 0.0033 Chi square).

### 3.3. Metastases

**Figure 2:**
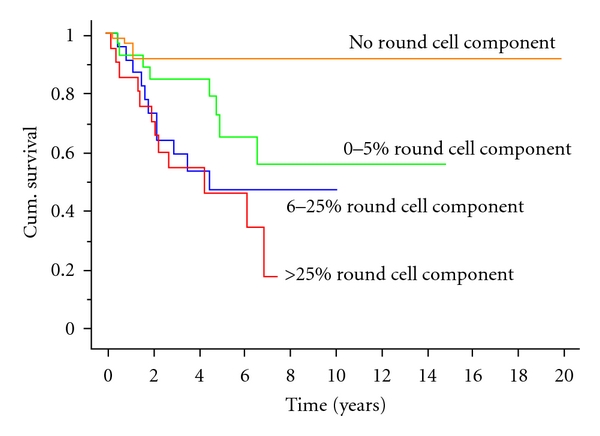
Kaplan Meier survival curve showing patient survival without development of metastatic disease split by round cell component.

52 patients (32.5%) developed metastases; the estimated risk of metastases developing was 34% at 5 years and 40% at ten years. The risk factors for development of metastases are shown in Table 2 and the time to development of these metastases with regard to the round cell component in Figure 2. In 7 patients the first site of metastases was in the lung alone, another 7 developed concomitant first metastases in the lung and another extrapulmonary site, and 38 patients developed extrapulmonary metastases alone. Among the extrapulmonary metastases, there was a wide variety of sites, with 5 patients having subcutaneous metastases, 8 developing limb muscle metastases, and 5 developing lymph node metastases. There were also 3 liver, 4 retroperitoneal, and 4 chest wall metastases with the remaining 9 patients developing multiple extrapulmonary sites of metastases. There was no clear correlation between the site of first metastases and the tumour size or round cell component of the primary tumours. When appropriate, metastases were surgically resected and some patients received adjuvant chemotherapy or radiotherapy. It was not infrequent for metastases to develop singly to start with and then further metastases to appear with increasing frequency [26]. Of the 52 patients who developed metastases, 38 had died at the time of last followup of progressive disease, with 2 dying of other causes. The median survival following diagnosis of metastases was 8.5 months (range 0 to 107 months). Survival following the diagnosis of metastases was not affected either by the site of the first metastases (lung or other) or by the round cell component. The 12 patients still alive following development of metastases have a followup ranging from 0 to 150 months (median 9.5 months).

### 3.4. Survival

**Figure 3:**
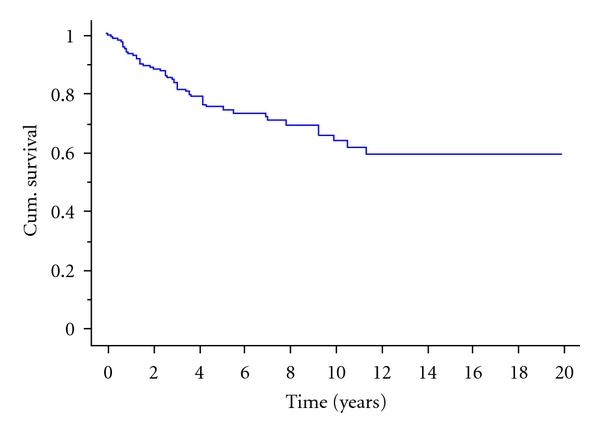
Disease-specific survival (all patients) with MRCLS.

**Figure 4:**
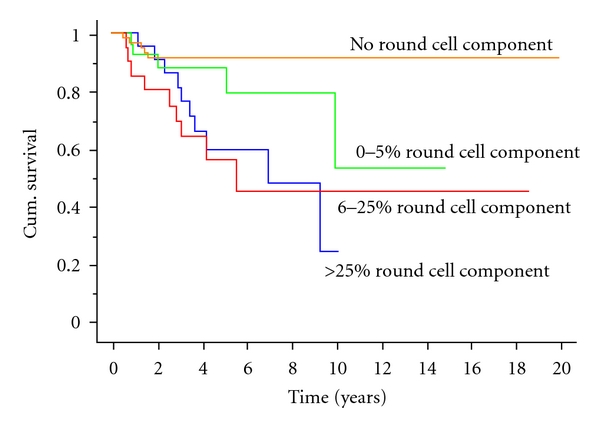
Disease-specific survival with regards to the round cell component of the tumours. It can be noted that extended follow-up time patients with tumours with 1–5% round cell component do less well than those with purely myxoid tumours while there is no apparent difference between patients with 6–25% round cell component and those with >25% round cell component.

Overall survival was 69% at 5 years, 56% at ten years, and 44% at 15 years. Disease-specific survival was 75% at 5 years, 63% at ten years, and 59% at 15 years (Figure 3). Factors affecting survival are shown in Table 3. The most significant factor affecting prognosis was the extent of the round cell component. Assessment of the significance of varying proportions of round cell component revealed that tumours with any round cell component (1–5% round cell component) carried a worse prognosis than those with no round cell component (Figure 4). This was, however, only apparent with increasing followup as there was little difference at 5 years but a dramatic difference at 10 years. The survival rate at ten years was 91% for those with no round cell component, 52% for those with up to 5% round cell component, and only 31% for those with >5% round cell component. A round cell component >5% carried the worst prognosis, but increasing levels of round cell component did not make any major difference to the prognosis, and the most obvious cut-off level was at 5% (Figure 3). On multivariate analysis, the round cell component remained a highly significant prognostic factor (Table 4). Interestingly both tumour size >10 cm and patient age >50 years were now significant poor prognostic factors as was amputation.

## 4. Discussion

This study reports on the clinical behavior and outcome in terms of local control, metastatic disease, and disease-specific survival in 160 patients with primary myxoid/round-cell liposarcoma, diagnosed and surgically treated at a single centre over the last 20 years. Our findings clearly identify that obtaining a good surgical margin is the most important single factor for maintaining local control. The single, most significant factor for metastatic disease and poor survival was the occurrence of a round cell component, which also increased the risk of local recurrence. 

In this series the risk of local recurrence was 12.9% at 5 years, which compares very favorably with other reported series. As expected there was a very strong correlation between risk of recurrence and margins achieved. 

In a more recent Mayo Clinic series of 95 cases, 14% developed local recurrence [8], and in a recent Dutch series of 49 cases the local recurrence rate was 33% [15]. Fiore et al. reported in a single centre study an overall risk of local recurrence of 21.7% at 5 years [14]. This series, however, included both primary and recurrent tumors; the recurrence rate for the primary tumors was almost identical to ours, with 11.3% local recurrence in patients with primary myxoid liposarcoma patients and 13.7% for those with round cell tumors.

The low local recurrence rate in their series of primary tumors was believed to be explained partly by the fact that almost half of the patients received postoperative radiotherapy. Interestingly, we achieved similarly low recurrence rates in our series in which 68% of all patients received adjuvant radiotherapy. 

Moreover, we found that the presence of a round cell component increases the risk of local recurrence by more than three times. Not surprisingly, the highest risk for local recurrence was seen in patients with tumours having >5% round cell component and involved or close margins. These are, therefore, the patients that should principally be considered for adjuvant treatment, certainly with radiotherapy and possibly chemotherapy. For patients with clear margins and a purely myxoid liposarcoma without round cell component, our recurrence rate was very low (4%, 2/53 patients). For this group the value of radiotherapy is probably more questionable. The lowest rate of local recurrence in patients with myxoid liposarcoma has been reported from the MD Anderson Cancer Center; only 3% of 127 patients, all of whom were treated with pre- or postoperative radiotherapy, developed local recurrence [27]. The somewhat surprising effectiveness of radiotherapy in these, for the most part, low-grade, slow-growing, and paucicellular tumours, is probably related to a radiation-induced lipogenic differentiation and cell cycle arrest [28]. It has also been suggested that the effectiveness of radiotherapy in myxoid liposarcoma is related to the radio-sensitivity of the delicate vasculature, characteristic of these tumours [29]. The occurrence of a local recurrence was in this series associated with an increased risk of developing metastases and tumour-related death. Thus, of the patients who had a local recurrence, 68% developed synchronous or subsequent metastases and 62% died of disease. Particularly early recurrences in liposarcoma in general have been found to indicate a poor prognosis [30]. Our findings show that this is also the case for the myxoid liposarcoma subtype. Whether the local recurrence is just a marker of a tumour with an aggressive behaviour or there is truly a causal association between local recurrence and metastasis, in other word if the local recurrence can be the source of metastases, remains, as for other soft tissue sarcomas, an open question. In any case, if such a causal association does exist, it is most likely weak compared to other risk factors [31, 32].

The generally accepted prognostic factors for soft tissue sarcomas are size, grade, and depth of the tumour, along with patient age [33]. The mean tumour size in this series was 11 cm, a similar figure to that in other series [8, 17, 28, 30]. Some of these studies have reported tumour size >10 cm to be associated with a poor prognosis, but, whilst we were unable to confirm this on univariate analysis, this was significant on multivariate analysis as was age over 50. The depth of the tumour was not significant in this series. This is even more surprising as we found that the median size of superficial tumours was 8 cm compared to a mean of 13 cm for deep tumours. There was no significant difference in size of tumours split by round cell component. 

The risk factors for development of metastases and indeed for overall survival are not surprisingly quite similar but are subtly different from those found in the generality of soft tissue sarcomas. Round cell component (and thus grade) is the only significant tumour factor whilst the need to perform an amputation is the only treatment factor, and this is likely to be a surrogate for a mixture of high-grade, large invasive tumours which may be expected to carry a poor prognosis. The overall disease-specific survival at 5 years of 91% for patients with pure myxoid liposarcomas and of 88% for those with <5% round cell component again compares favourably with those reported by others. Fiore et al. reported a 93% disease-specific survival for patients with myxoid liposarcoma which included all patients with up to 5% round cell component [14]. However, Fiore's results for patients with >5% round cell component of 87% five-year survival are dramatically better that those in our experience, where patients had only a 58% survival at 5 years. The reason for this is not clear as Fiore et al. reported a 21.7% risk of metastases at 5 years for this group compared to our 54% risk. The difference in metastatic risk could be explained by the use of adjuvant chemotherapy as this was used in 39 of 177 patients in Fiore's series compared to none in ours. Unfortunately it is not clear which patients received this, but presumably those at greatest perceived risk (round cell tumours) were selected (and this has since been confirmed (personal communication). Dalal et al. have, however, followed a very similar treatment protocol to us and reported a 92% five years disease-specific survival for patients with less than 5% round cell component compared to a 74% five years disease-specific survival for patients with more than 5% round cell component [34]. 

We have also confirmed other opinions about the cutoff of 5% round cells as being a watershed between intermediate- and high-risk tumours. We have, however, shown that an increasing % of round cells does not confer extra risk, and we have also shown that even patients with <5% round cells are at greater risk of both metastases and death than those with pure myxoid liposarcomas. Indeed, pure myxoid liposarcoma with no round cell component has a very good prognosis in our series with no tumour-related death reported after the first 18 months. 

The role of adjuvant chemotherapy for patients with soft tissue sarcomas has been extensively investigated [35], and recent work has highlighted the potential sensitivity of myxoid liposarcomas to chemotherapy [18, 19, 27, 36]. Given the high rate of metastatic disease developing in patients with >5% round cell component, we consider this group of patients to be at particular risk and suitable for future trials of drug therapy. If patients develop round cell features at the time of recurrence, then this also indicates a change to a higher grade and the possible need for more aggressive treatment [37].

Like others, we have found a high risk of metastases developing outside the lungs. In our series only 19% of first metastases were in the lung, and in half of these patients other sites were identified on staging. 81% of first metastases were, thus, extrapulmonary but in a wide range of sites. There was no obvious predictive factor we could identify that predicted whether a patient would develop pulmonary or extrapulmonary metastases. We have only analysed the location of the first metastases but noted the sometime prolonged course of patients who develop extrapulmonary metastases at a multitude of sites [26]. Estourgie found extrapulmonary metastases in 12 of 22 patients with metastatic disease (55%) and recommended that patients should be followed with regular abdominal/pelvic CT scans [13]. If this had been carried out in our patients, it would have detected the 14 patients with pulmonary metastases (all of whom had their metastases identified by routine surveillance chest radiograph) but only 13 of the other 38 (34%) metastases, possibly before they became symptomatic.

 Cheng et al. found that patients with extremity liposarcoma when metastasized had a 59% incidence of isolated extrapulmonary metastasis [11], whilst Guadagnolo et al. reported that 78% of all their metastases were extrapulmonary and 48% were retroperitoneal [27]. Others have also noted the high incidence of purely extrapulmonary metastases, Fiore et al. reporting a rate of 41% [14], Ten Heuvel et al. a rate of 77% [15], and Antonescu et al. a rate of 67% [16]. All of these series noted that common sites of metastasis were the retroperitoneum, abdominal or chest wall, and abdominal cavity whilst Schwab et al. noted the presence of skeletal metastases [38].

The question of how to follow up these patients has not yet been answered. There are several reports of the failure of both PET scans and bone scans to detect myxoid liposarcoma metastases [39, 40]. Other options of whole body CT or MRI remain as possible screening tools, but there is lack of proof for both clinical and cost effectiveness. If we were to add some form of whole-body imaging (e.g., whole-body MRI) to our current protocol of clinical followup and chest radiograph, this may have detected 38 patients with metastases at extrapulmonary sites before they became symptomatic. Assuming that these metastases would have been detected on average three months earlier by routine imaging than they would have been by just clinical followup would have meant that a total of 1578 scans would have been done to detect 30 extrapulmonary metastases in the first five years following diagnosis, that is, 52 scans for each metastasis detected. If the scans were only done with half that frequency, then some of the metastases may have become apparent in between scans, but, assuming this was not the case, it would still mean 26 scans for each metastasis detected. If only patients with a high round cell (>5%) component were investigated; however, the figures are more reasonable, with 17 extrapulmonary metastases being potentially identified after 370 scans in the first five years, that is, 22 scans for each metastasis detected. Whether earlier detection of these metastases would influence outcome, however, remains unclear and is a topic worthy of further investigation. 

In conclusion we have confirmed that the round cell component is the most significant predictor of behaviour of the tumour for myxoid liposarcoma. Pure myxoid liposarcoma is a relatively low-grade tumour which should be treated surgically with attempts to obtain clear margins of excision. Patients with 0–5% round cell component represent an intermediate-grade tumour with a higher risk of metastatic disease, whilst patients with >5% round cell component must be considered to have a high-grade tumour with a significantly increased risk of both local recurrence and metastases and should be treated with surgical excision with clear margins, radiotherapy, and possibly chemotherapy. The use of new agents may well be indicated for this patient group. Awareness of the high incidence of extrapulmonary metastases should lead to investigation of a suitable clinical and cost-effective method of screening for these, and this may be particularly important if effective new treatments for metastatic disease become available.

## Figures and Tables

**Table 1:** Risk factors for local recurrence at 5 years following tumour resection with hazards ratio estimates, 95% confidence limits and *P* values.

Factor	5 years risk of LR	HR	95% CI	*P* value
*Overall*	*13%*			

Depth:				
Superficial	9%	1.46	0.42–5.00	0.55
Deep	14%	1		

Size:				
Up to 5 cm	50%	2.285	0.63–8.27	0.208
>5 cm to <10 cm	7%	0.681	0.24–1.96	0.476
>10 cm	14%	1		

Site:				
Lower extremity	12%	0.269	0.06–1.18	0.081
Upper extremity	40%	1		
Trunk	20%	0.605	0.60–6.76	0.683

Histology:				
Pure myxoid	9%	1		
0–5% round cell	4%	0.472	0.055–4.04	0.493
>5% round cell	26%	3.321	1.12–9.82	0.03

Margin:				
Intralesional	34%	2.445	0.85–7.06	0.098
Marginal	6%	0.395	0.13–1.21	0.103
Wide	15%	1		

Surgery:				
Amputation	10%	0.625	0.083–4.69	0.648
Limb salvage	13%	1		

*Postoperative radiotherapy*	11%	1		
No radiotherapy	17%	1.13	0.429–2.977	0.80

**Table 2:** Factors leading to development of metastatic disease and risk of developing this within 5 years, showing hazards ratio estimates, 95% confidence limits and *P* values.

Factor	5 years risk of metastases	HR	95% CI	*P* value
*Overall*	*34%*			

Depth:				
Superficial	28%	1		
Deep	37%	1.443	0.68–3.07	0.341

Size:				
Up to 5 cm	22%	0.771	0.24–2.52	0.667
> 5 cm to <10 cm	28%	0.69	0.38–1.27	0.232
>10 cm	38%	1		

Site:				
Lower extremity	33%	0.889	0.22–3.66	0.871
Upper extremity	40%	1		
Trunk	20%	0.780	0.07–8.68	0.84

Histology:				
Pure myxoid	8%	1		
0–5% round cell	35%	4.166	1.36–12.78	0.0126
>5% round cell	54%	8.899	3.35–23.64	<0.0001

Margin:				
Intralesional	55%	1.347	0.61–2.96	0.459
Marginal	26%	0.722	0.39–1.31	0.286
Wide	37%	1		

Surgery:				
Amputation	73%	3.091	1.546–6.179	0.0014
Limb salvage	29%	1		

*Postoperative radiotherapy*	28%	0.725	0.38–1.38	0.329
No radiotherapy	36%	1		

**Table 3:** Factors affecting disease specific survival, showing hazards ratio estimates, 95% confidence limits and *P* values.

Factor	5 years disease specific survival	HR	95% CI	*P* value
*Overall*	*75%*			

*Age under 50*	81%	0.543	0.36–1.12	0.139
Age over 50	66%	1		

Depth:				
Superficial	87%	1		
Deep	71%	1.69	0.72–3.99	0.232

Size:				
Up to 5 cm	73%	0.647	0.15–2.71	0.552
> 5 cm to <10 cm	78%	0.607	0.31–1.18	0.143
>10 cm	72%	1		

Site:				
Lower extremity	76%	0.793	0.19–3.29	0.749
Upper extremity	60%	1		
Trunk	50%	1.142	0.10–12.89	0.914

Histology:				
Pure myxoid	91%	1		
0–5% round cell	88%	2.785	0.80–9.69	0.107
>5% round cell	58%	6.897	2.53–18.82	0.0002

Margin:				
Intralesional	70%	1.493	0.65–3.46	0.34
Marginal	78%	0.721	0.38–1.38	0.32
Wide	71%	1		

Surgery:				
Amputation	51%	3.062	1.47–6.37	0.0027
Limb salvage	77%	1		

*Postoperative radiotherapy*	76%	1		
No radiotherapy	74%	0.975	0.50–1.89	0.975

**Table 4:** Multivariate analysis showing factors.

Factor	HR	95% CI	*P* value
*Age under 50*	0.415	0.190–0.908	0.0276
*Size over 10 cm*	2.635	1.095–6.339	0.0305

Histology:			
Pure myxoid	1		
0–5% round cell	3.504	0.994–12.345	0.0510
>5% round cell	9.825	3.505–27.542	<0.0001

Surgery:			
Amputation	3.271	1.161–9.221	0.0250
Limb salvage	1		
